# The InterPro protein families and domains database: 20 years on

**DOI:** 10.1093/nar/gkaa977

**Published:** 2020-11-06

**Authors:** Matthias Blum, Hsin-Yu Chang, Sara Chuguransky, Tiago Grego, Swaathi Kandasaamy, Alex Mitchell, Gift Nuka, Typhaine Paysan-Lafosse, Matloob Qureshi, Shriya Raj, Lorna Richardson, Gustavo A Salazar, Lowri Williams, Peer Bork, Alan Bridge, Julian Gough, Daniel H Haft, Ivica Letunic, Aron Marchler-Bauer, Huaiyu Mi, Darren A Natale, Marco Necci, Christine A Orengo, Arun P Pandurangan, Catherine Rivoire, Christian J A Sigrist, Ian Sillitoe, Narmada Thanki, Paul D Thomas, Silvio C E Tosatto, Cathy H Wu, Alex Bateman, Robert D Finn

**Affiliations:** European Molecular Biology Laboratory, European Bioinformatics Institute (EMBL-EBI), Wellcome Genome Campus, Hinxton, Cambridgeshire CB10 1SD, UK; European Molecular Biology Laboratory, European Bioinformatics Institute (EMBL-EBI), Wellcome Genome Campus, Hinxton, Cambridgeshire CB10 1SD, UK; European Molecular Biology Laboratory, European Bioinformatics Institute (EMBL-EBI), Wellcome Genome Campus, Hinxton, Cambridgeshire CB10 1SD, UK; European Molecular Biology Laboratory, European Bioinformatics Institute (EMBL-EBI), Wellcome Genome Campus, Hinxton, Cambridgeshire CB10 1SD, UK; European Molecular Biology Laboratory, European Bioinformatics Institute (EMBL-EBI), Wellcome Genome Campus, Hinxton, Cambridgeshire CB10 1SD, UK; European Molecular Biology Laboratory, European Bioinformatics Institute (EMBL-EBI), Wellcome Genome Campus, Hinxton, Cambridgeshire CB10 1SD, UK; European Molecular Biology Laboratory, European Bioinformatics Institute (EMBL-EBI), Wellcome Genome Campus, Hinxton, Cambridgeshire CB10 1SD, UK; European Molecular Biology Laboratory, European Bioinformatics Institute (EMBL-EBI), Wellcome Genome Campus, Hinxton, Cambridgeshire CB10 1SD, UK; European Molecular Biology Laboratory, European Bioinformatics Institute (EMBL-EBI), Wellcome Genome Campus, Hinxton, Cambridgeshire CB10 1SD, UK; European Molecular Biology Laboratory, European Bioinformatics Institute (EMBL-EBI), Wellcome Genome Campus, Hinxton, Cambridgeshire CB10 1SD, UK; European Molecular Biology Laboratory, European Bioinformatics Institute (EMBL-EBI), Wellcome Genome Campus, Hinxton, Cambridgeshire CB10 1SD, UK; European Molecular Biology Laboratory, European Bioinformatics Institute (EMBL-EBI), Wellcome Genome Campus, Hinxton, Cambridgeshire CB10 1SD, UK; European Molecular Biology Laboratory, European Bioinformatics Institute (EMBL-EBI), Wellcome Genome Campus, Hinxton, Cambridgeshire CB10 1SD, UK; European Molecular Biology Laboratory, Structural and Computational Biology Unit, Meyerhofstraße 1, 69117 Heidelberg, Germany; Swiss-Prot Group, Swiss Institute of Bioinformatics, CMU, 1 rue Michel Servet, CH-1211, Geneva 4, Switzerland; Medical Research Council Laboratory of Molecular Biology, Cambridge Biomedical Campus, Francis Crick Ave, Trumpington, Cambridge CB2 0QH, UK; National Center for Biotechnology Information, National Library of Medicine, National Institutes of Health, 8600 Rockville Pike, Bethesda MD 20894 USA; Biobyte Solutions GmbH, Bothestr 142, 69126 Heidelberg, Germany; National Center for Biotechnology Information, National Library of Medicine, National Institutes of Health, 8600 Rockville Pike, Bethesda MD 20894 USA; Division of Bioinformatics, Department of Preventive Medicine, University of Southern California, Los Angeles, CA 90033, USA; Protein Information Resource, Georgetown University Medical Center, Washington, DC 20007, USA; Department of Biomedical Sciences, University of Padua, via U. Bassi 58/b, 35131 Padua, Italy; Department of Structural and Molecular Biology, University College London, Gower St, Bloomsbury, London WC1E 6BT, UK; Medical Research Council Laboratory of Molecular Biology, Cambridge Biomedical Campus, Francis Crick Ave, Trumpington, Cambridge CB2 0QH, UK; Swiss-Prot Group, Swiss Institute of Bioinformatics, CMU, 1 rue Michel Servet, CH-1211, Geneva 4, Switzerland; Swiss-Prot Group, Swiss Institute of Bioinformatics, CMU, 1 rue Michel Servet, CH-1211, Geneva 4, Switzerland; Department of Structural and Molecular Biology, University College London, Gower St, Bloomsbury, London WC1E 6BT, UK; National Center for Biotechnology Information, National Library of Medicine, National Institutes of Health, 8600 Rockville Pike, Bethesda MD 20894 USA; Division of Bioinformatics, Department of Preventive Medicine, University of Southern California, Los Angeles, CA 90033, USA; Department of Biomedical Sciences, University of Padua, via U. Bassi 58/b, 35131 Padua, Italy; Protein Information Resource, Georgetown University Medical Center, Washington, DC 20007, USA; European Molecular Biology Laboratory, European Bioinformatics Institute (EMBL-EBI), Wellcome Genome Campus, Hinxton, Cambridgeshire CB10 1SD, UK; European Molecular Biology Laboratory, European Bioinformatics Institute (EMBL-EBI), Wellcome Genome Campus, Hinxton, Cambridgeshire CB10 1SD, UK

## Abstract

The InterPro database (https://www.ebi.ac.uk/interpro/) provides an integrative classification of protein sequences into families, and identifies functionally important domains and conserved sites. InterProScan is the underlying software that allows protein and nucleic acid sequences to be searched against InterPro's signatures. Signatures are predictive models which describe protein families, domains or sites, and are provided by multiple databases. InterPro combines signatures representing equivalent families, domains or sites, and provides additional information such as descriptions, literature references and Gene Ontology (GO) terms, to produce a comprehensive resource for protein classification. Founded in 1999, InterPro has become one of the most widely used resources for protein family annotation. Here, we report the status of InterPro (version 81.0) in its 20th year of operation, and its associated software, including updates to database content, the release of a new website and REST API, and performance improvements in InterProScan.

## INTRODUCTION

Recent advances in genomic technologies combined with the substantial reductions in the cost of sequencing have enabled the scientific community to generate new sequencing data at an unprecedented scale. With entire genomes being routinely sequenced, and environmental samples yielding hundreds of millions of sequences, there is a crucial need to classify proteins that have not been, and likely never will be experimentally characterized. To address this challenge, several automated sequence analysis methods have been developed to annotate protein families, domains, and functional sites by transferring the information from an experimentally characterized sequence to uncharacterized sequences using predictive diagnostic models (hidden Markov models, patterns, profiles or fingerprints), known as *signatures*. A number of protein signature databases have been developed, each having their own field of interest (e.g. protein superfamilies, functional and structural domains, orthologous groups). InterPro integrates 13 protein signature databases into one central resource: CATH-Gene3D (1), the Conserved Domains Database (CDD) (2), HAMAP (3), PANTHER (4), Pfam (5), PIRSF (6), PRINTS (7), PROSITE Patterns (8), PROSITE Profiles (8), SMART (9), the Structure–Function Linkage Database (SFLD) (10), SUPERFAMILY (11) and TIGRFAMs (12). Collectively, member databases provide complementary levels of protein annotation, making InterPro a comprehensive resource about protein families, domains, and functional sites.

InterPro also offers additional annotations on sequence features such as intrinsic protein disorder regions (provided by MobiDB-lite, part of the MobiDB database (13)), and signal peptides, transmembrane regions and coiled-coils (provided by Coils (14), Phobius (15), SignalP (16) and TMHMM (17)).

Where signatures from two or more member databases represent the same biological entity, the member database signatures are integrated together into one InterPro entry, reducing redundancy. Member database signatures that are integrated into Interpro are carefully checked by curators prior to integration. InterPro entries are annotated with a unique name and InterPro accession number, a descriptive abstract and Gene Ontology (GO) terms (18) that can be consistently assigned to all proteins matched by that entry. An entry type (family, domain, repeat, site or homologous superfamily) is also assigned.

The majority of member databases use single signatures to represent families, domains, repeats and sites, and consequently their sequence matches do not usually change significantly over time. Where a domain or family matches a subset of sequences matched by another domain or family respectively, hierarchical relationships are indicated (e.g. child and parent). These hierarchical relationships allow InterPro to provide increasingly detailed levels of functional information on proteins. The hierarchical relationships are type specific, i.e. domain entries can only be placed in a hierarchy with other domains, not with families, and vice versa.

The CATH-Gene3D and SUPERFAMILY databases use collections of underlying HMMs per entry, which represent diverse structural families. Additional HMMs may be added to such entries as new related, but diverse, structures are determined. The ‘homologous superfamilies’ entry type is exclusively used for CATH-Gene3D and SUPERFAMILY entries to accommodate the increased flux in the sequences matched by entries from these two databases over time. For the same reason, homologous superfamilies are not manually curated into hierarchies, but instead, their relationships to other InterPro entries are calculated automatically based on the intersection of their matched sequence set. The Jaccard similarity index and Jaccard containment index are evaluated for each pair of homologous superfamily and InterPro entry, and if either of these indices is ≥0.75, it is assumed that the members of the pair are related to each other.

Not only are all new entries manually curated, but the entire InterPro database is regularly reviewed for accuracy. Notably InterPro routinely incorporates new member database releases. For every signature in the new member database release (both new and pre-existing) matches from the latest version of UniprotKB are determined. For any pre-existing signatures for which the matched sequences have changed, the InterPro entry is checked for false positive matches (where a signature returns incorrect matches). These entries are reviewed and updated or removed where necessary. Where possible, new signatures are integrated into existing or new InterPro entries. The incorporation of member database new releases is a key time in which new signatures are integrated into InterPro.

In addition to specific member database updates, signatures from all member databases are reviewed at each InterPro release cycle. Matches to the latest monthly release of UniprotKB/Swiss-Prot are calculated and any signature for which the retrieved matches have altered is manually reviewed. This alerts us for example when the function of a previously uncharacterised protein becomes known allowing us to rapidly update the entry.

InterPro's GO annotation is based both on the experimental evidence available for characterized proteins and the taxonomic range of proteins matched by a particular InterPro entry (e.g. plant-specific GO terms cannot be added to an InterPro entry that matches proteins from organisms other than plants). Once a GO term is applied to an InterPro entry, it is automatically propagated to all the UniProtKB proteins matched by that entry (19). This process enables the transfer of GO-based functional information from relatively few experimentally characterized sequences to a set of evolutionarily related but as yet uncharacterized sequences. InterPro curators receive regular feedback through the GO-annotation tracking system, which is used by members of the GO community to highlight misannotations and to suggest annotation improvements.

The significant level of expert curation undertaken for both new and existing entries, and the use of different entry types enables InterPro protein classification to keep up with the ever-increasing amount of protein sequence, structure and member database signature data available.

## RESULTS

### Training materials and documentation

Following the release of the new InterPro website, the InterPro online materials have been updated and made available on the new EBI training platform (https://www.ebi.ac.uk/training/online/) to support the new and updated features (e.g. browse feature, sequence viewer). We also organised a series of four webinars:

- Understanding InterPro families, domains and functions: explains what InterPro is to our new users,
- Using the InterPro website in your research: presents the different ways the new website can be queried,
- Accessing InterPro programmatically: explains how to use the InterPro API for bioinformatics analysis,
- InterProScan: presents the software underlying the InterPro sequence search and how it can be used locally by users.

As part of the InterPro training material, the recording of the webinars are freely available on the EBI training platform. We have also expanded the InterPro documentation and moved it into the Read the Docs platform (https://interpro-documentation.readthedocs.io/en/latest/). Read the Docs, provides a web based version making the content easy to navigate through, as well as providing it in a variety of different formats (PDF, HTML, Epub).

### Content updates

Like UniProtKB, InterPro follows an 8-week release cycle. Each InterPro release contains new entries, created by integrating member database signatures, and may include one or more member database updates. Since our previous publication that described InterPro 70.0 in 2018 (20), there have been 12 InterPro releases, integrating 10 member database updates: CDD (3.17), HAMAP (2019_01, 2020_01), PANTHER (14.1), Pfam (32.0, 33.1), PROSITE Patterns (2019_01, 2019_11) and PROSITE Profiles (2019_01, 2019_11).

ProDom is a database of protein domain families based on the automatic clustering of sequences by similarity (21). Because it has not been updated since December 2015, we removed ProDom from InterPro in release 74.0. In recent years, ProDom signatures were only integrated when there was a correspondence with at least another signature, so removing ProDom did not result in the loss of many InterPro entries: out of the 1310 entries integrating a ProDom signature in release 73.0, 1165 entries were still available in release 74.0. The TIGRFAMs database was transferred in April 2018 from the J. Craig Venter Institute (JCVI, formerly known as The Institute for Genomic Research, TIGR) to the National Center for Biotechnology Information (NCBI), where it is now actively maintained within the set of HMMs used in RefSeq annotation (22).

Over the past two years, 1558 member database signatures have been integrated into existing InterPro entries, and 3315 have contributed to the creation of 3280 new entries.

Considering the entries that have been deleted, InterPro version 81.0 consists of 37 821 entries based on 50 637 integrated member database signatures. As a consequence, the InterPro coverage of sequences in UniProtKB (i.e. the proportion of proteins with one or more InterPro annotations) increased from 80.9% (InterPro version 70.0) to 81.3% (InterPro version 81.0, see Table 1). Although a 0.4% increase may seem small, we should consider that UniProtKB considerably grew in the same period (from ∼125 million sequences to ∼189 million). Therefore, the small increase in InterPro's coverage represents ∼50 million additional sequences with at least one InterPro annotation. We previously reported that 78.4% of sequences in the UniProt Archive (UniParc) were annotated by InterPro (23). During the last four years, this coverage increased to 80.3%.

**Table 1.** Coverage of UniProtKB and UniParc (non-redundant archive of protein sequences) by InterPro entries (version 81.0)

Protein sequence database	Number of sequence entries	Number of sequences entries with one or more matches to InterPro
UniProtKB/reviewed	563 082	544 375 (96.7%)
UniProtKB/unreviewed	188 961 949	153 602 947 (81.3%)
UniProtKB (total)	189 525 031	154 147 322 (81.3%)
UniParc	340 835 553	273 569 610 (80.3%)

InterPro regularly incorporates member database updates, which provides a constant stream of new signatures for integration. However, the overall integration figures often hide a lot of curation work. Updating member databases remains a challenge, especially when it involves substantial data changes. For example, many InterPro entries were at risk of being deleted when updating PANTHER to version 14.1, because the underlying signatures had been either changed or deleted as part of the rebuild process between PANTHER 12.0 and 14.1. The percentage of member database signatures integrated into InterPro for each member database is shown in Table 2. The level of integration is over 90% for all databases, except CATH-Gene3D, CDD, PANTHER, SFLD and SUPERFAMILY. CATH-Gene3D and SUPERFAMILY are the databases that InterPro primarily relies on for broad and diverse domain families. As CATH-Gene3D and SUPERFAMILY use a different methodology from other InterPro's member databases, in that they rely on a collection of underlying HMMs to represent diverse structural families rather than one single model, their signatures can only be integrated in a new type of InterPro entries: *homologous superfamilies*. The diversity of signatures provided by CATH-Gene3D and SUPERFAMILY, along with their relative lack of annotations, makes their integration in InterPro challenging. However, we are working in close collaboration with these databases to help them improve their annotation and hence increase their integration in InterPro. PANTHER provides ∼120 000 signatures, which is more than twice as many as other member databases. Such a backlog of signatures will take a considerable amount of curation time. In addition, PANTHER updates involve a large number of deleted or changed signatures, potentially resulting in a large number of lost integrated signatures. Finally, CDD and SLFD are the most recent additions to InterPro (incorporated in InterPro 58.0 and 59.0, respectfully). CDD is a collection of almost 15 000 conserved domain footprints in version 3.17, which is ∼2000 signatures more than in version 3.14 (included in InterPro version 70.0), and SFLD, a smaller scale database, describes structure-function relationships for functionally diverse enzyme superfamilies. Both CDD and SFLD provide hierarchical classifications, but their hierarchies differ from InterPro's classification, limiting their integration.

**Table 2.** Release version and number of member database signatures integrated into InterPro version 81.0

Member database	Release number	Total signatures	Integrated signatures
CATH-Gene3D	4.2.0	6119	2646 (43.2%)
CDD	3.17	14 908	3211 (21.5%)
HAMAP	2020_01	2327	2325 (99.9%)
PANTHER	14.1	123 151	9569 (7.8%)
Pfam	33.1	18259	17400 (95.3%)
PIRSF	3.10	3285	3233 (98.4%)
PRINTS	42.0	2106	1950 (92.6%)
PROSITE patterns	2019_11	1311	1286 (98.1%)
PROSITE profiles	2019_11	1265	1172 (92.6%)
SFLD	4	303	147 (48.5%)
SMART	7.1	1312	1264 (96.3%)
SUPERFAMILY	1.75	2019	1618 (80.1%)
TIGRFAMs	15	4488	4431 (98.7%)

**Figure 1.**
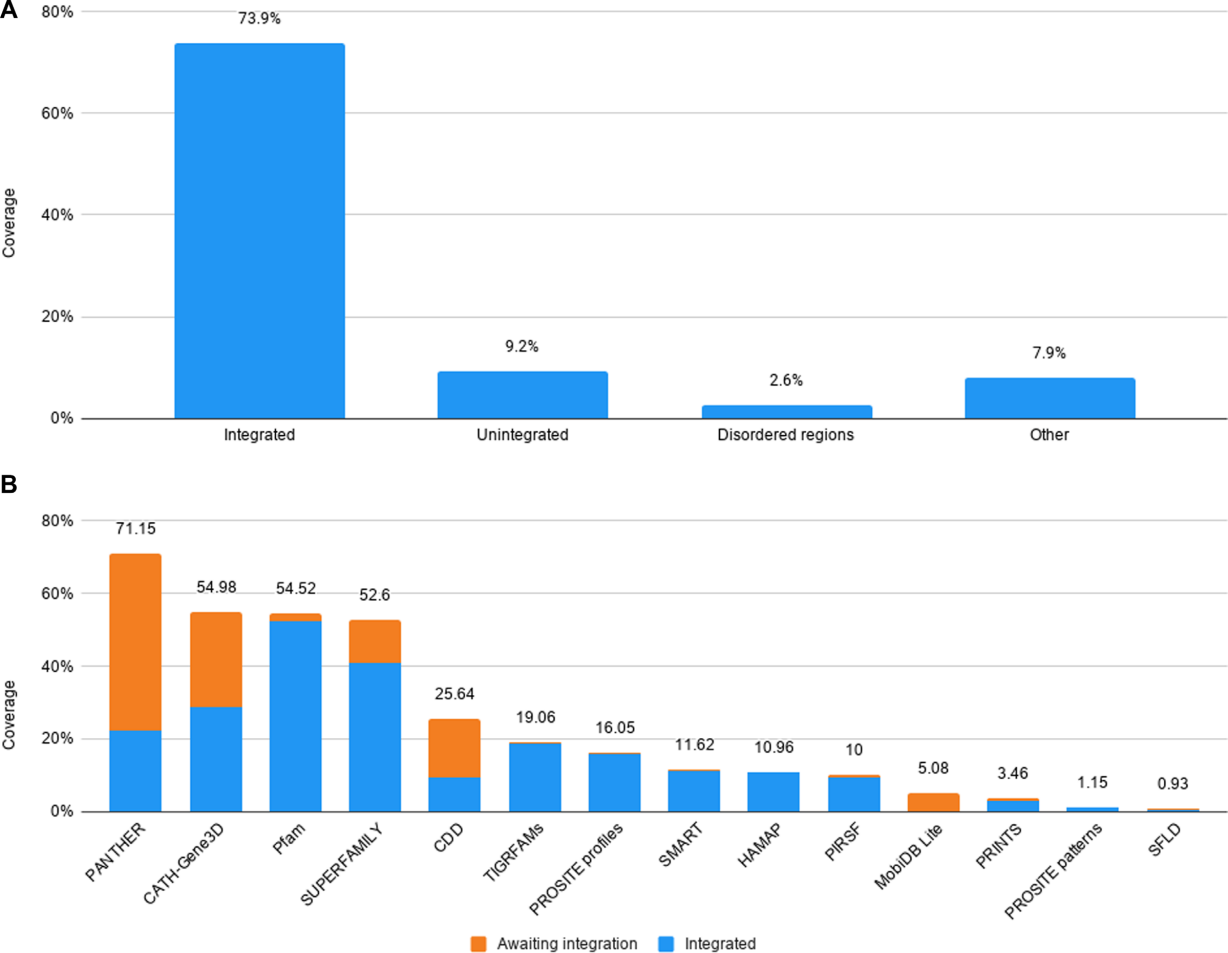
InterPro coverage of amino acid residues in UniProtKB. (**A**) Unique residue coverage of UniProtKB by signatures integrated into InterPro, member database signatures awaiting integration, intrinsically disordered regions, and other regions predicted to be signal peptides, transmembrane domains or coiled-coils. (**B**) Residue coverage of InterPro's contributing member databases. Residues matched by signatures integrated into InterPro are shown in blue, and residues found only in signatures not yet integrated are shown in orange.

In addition to InterPro's sequence coverage, we also evaluated the amino acid residue coverage of InterPro and its member databases. As of InterPro version 81.0, 73.9% of residues found in UniProtKB are annotated by InterPro entries, and 9.2% are annotated by signatures awaiting integration only. When no annotation from InterPro and member databases is available, residues can still benefit from some levels of annotations: 2.6% of residues are found in intrinsically disordered regions. Finally, 7.9% of residues are only found in other sequence features such as coiled-coils, transmembrane regions and signal peptides. The cumulative unique residue coverage is shown in Figure 1A. Overall, 93.6% of UniProtKB residues (58.4 billion) receive some level of annotation, leaving only 6.4% that are yet to be covered by the InterPro consortium.

We then focused on the residue coverage of member databases to evaluate their individual contributions to the InterPro coverage of residues in UniProtKB (Figure 1B). PANTHER provides the greatest coverage, which may be expected as it is the largest member database in terms of signatures, and because its families are built for full-length protein sequences. Although fewer than 10% of PANTHER signatures are integrated in InterPro, 32% of UniProtKB residues annotated by PANTHER are hit by PANTHER signatures integrated in InterPro. This is because the largest PANTHER families are integrated into InterPro. PANTHER also provides the greatest unique coverage of specific residues not in InterPro (6.4%), i.e. residues that are only covered by PANTHER families yet to be integrated. Focussing the curation effort on these families will maximize the contribution of PANTHER to InterPro. CATH-Gene3D, Pfam, and SUPERFAMILY offer comparable levels of residue coverage, but lower than PANTHER’s as they contain fewer models and focus on domains. Similarly to PANTHER, just over half (53%) of residues annotated by CATH-Gene3D are matched by a CATH-Gene3D signature integrated in InterPro, while under half (43%) of CATH-Gene3D signatures are integrated.

### COVID-19 updates

Following the emergence of the COVID-19 disease, we have reviewed and updated existing annotations for InterPro entries related to the SARS-CoV-2 proteome (UniProt Proteome Identifier: UP000464024) and delivered a partial InterPro release (InterPro 78.1, 7 April 2020), including easy access to the annotations from the InterPro homepage. In addition to our standard review procedures, for the partial InterPro release 78.1 steps were taken specifically for the SARS-CoV-2-related entries as described below.

Firstly we performed an analysis to identify all InterPro entries and existing member database signatures that matched any of the SARS-CoV-2 proteins. All SARS-CoV-2-related InterPro entries were reviewed, even those for which there were no changes in the proteins matched by the signature(s). Written abstracts for these entries were updated to reflect recent published research findings. Specific information was also added to the written abstracts regarding the importance of the entry (protein family, domain or site) to SARS coronaviruses.

The names used for SARS-CoV-2 related entries were also updated to provide consistent and accurate nomenclature. Due to ribosomal frameshifting the SARS-CoV-2 genome encodes two large, replicase polyproteins (ORF1a and ORF1ab). These polyproteins are proteolytically cleaved into non-structural proteins that assemble into a large membrane bound replication-transcription complex (RTC). In InterPro, we use the prefix NSP- to refer to the non-structural proteins encoded by the replicase polyproteins. Following ORF1a/ORF1ab, the SARS-CoV-2 genome encodes 4 structural proteins (spike (S), envelope (E), membrane (M) and nucleocapsid (N)) interspersed with accessory proteins (which are usually called non-structural accessory proteins, although some of them constitute structural parts of the virion). We use the prefix NS- to refer to the individually encoded non-structural accessory proteins.

Lastly, we identified any previously unintegrated member database signatures related to SARS-CoV-2 that could now be integrated as a result of changes in the proteins matched by the signature. Eleven new InterPro entries related to SARS-CoV-2 were created during this process, including homologous superfamily entries for the coronavirus spike glycoprotein S2 (IPR043473), the macro domain-like (IPR043472), the NSP3, SUD-N (Mac2) domain (IPR043478) and Peptidase C30, domain 3 (IPR043477). The spike glycoprotein (S) of coronaviruses is essential for viral entry, with the membrane-anchored S2 subunit mediating fusion of the viral and host cell membranes. The macro-domain like homologous superfamily includes members from a wide range of species including coronaviruses. The coronavirus (CoV) macro-domain (MAC1) is present in non-structural protein 3 (NSP3) and binds to and removes ADP-ribose adducts from proteins. A homologous superfamily was also created for MAC2. In contrast to MAC1, the MAC2 and MAC3 domains are unique macrodomains present in NSP3 from SARS-CoV and SARS-CoV-2 viruses. Peptidase C30 (NSP5) is the main protease in coronaviruses and is responsible for cleavage of the coronavirus replicase polyprotein into individual functional proteins. The third of the three peptidase C30 domains, Domain III has been implicated in the proteolytic activity of this crucial enzyme.

Through the steps described above, all SARS-CoV-2 related InterPro entries, both new and pre-existing, were carefully reviewed and updated appropriately prior to inclusion in the partial Interpro release 78.1.

Furthermore, as a result of the COVID-19 pandemic, the Pfam member database undertook a review of all Pfam signatures related to SARS-CoV-2 and generated new Pfam signatures to increase coverage of the SARS-CoV-2 proteome. Pfam 33.1 was released in May 2020 and included the updated SARS-CoV-2 related signatures. Flat files of the SARS-CoV-2 related signatures from Pfam 33.1 were made available on the InterPro website while a curation effort got underway to integrate Pfam 33.1 into InterPro 80.0 following our standard curation procedures. The additional curation steps described above were again taken for the SARS-CoV-2 related signatures.

During integration of Pfam 33.1, 10 InterPro entries were created for new SARS-CoV-2 related Pfam signatures. These included domain entries for the following replicase polyprotein non-structural proteins: NSP2 (N- and C-terminal domains), NSP3 (C-terminal), NSP4 (N-terminal), and NSP15 (N-terminal oligomerisation, middle, and uridylate specific endoribonuclease). Following cleavage of the replicase polyprotein, these NSPs all assemble into the replication-transcription complex, which is essential for the synthesis of viral RNA. For several of these domains, more specific structural and functional information is available. For example, NSP4 is a membrane-spanning protein that interacts with NSP3. The N-terminal domain of coronavirus NSP4 (IPR043612) represents the four membrane spanning regions. NSP15 is an RNA uridylate-specific endoribonuclease. The N-terminal domain (IPR043606) is critical for formation of hexamers thought to be the functional unit of NSP15. The function of the NSP15 middle domain (IPR043608) is not yet known, however the NSP15 C-terminal domain (IPR043609) contains the active site of the enzyme. NSP6 is a membrane protein containing six transmembrane domains with a large C-terminal tail and an InterPro family entry (IPR043610) was created to integrate the NSP6 Pfam signature. New domain entries were also created to represent the Coronavirus Spike S1 subunit (IPR002551), which is responsible for receptor binding, and the cysteine rich intravirion region found at the C-terminus of the Coronavirus Spike S2 subunit (IPR043614).

Interpro relies on the invaluable contributions of its member databases. In response to the COVID-19 outbreak we have sought to adapt our procedures to provide up-to-date classification for SARS-CoV-2 related protein sequences and have created an easy route to access this information. InterPro currently contains over 70 entries related to SARS-CoV-2, which include protein families, domains, sites, and homologous superfamilies and together cover the majority of the SARS-CoV-2 proteome. Due to our continuous curation process as new sequence matches to SARS-Cov-2 signatures are identified and new signatures are created by member databases, the InterPro database will be updated appropriately to provide timely classification of SARS-CoV-2 proteins to the global research community.

### Website and REST API

We announced the new InterPro website in the previous NAR paper (20) as a beta release; we have now released it as the main InterPro website. Additionally, since our last publication, we have introduced many improvements to the website both to improve ease of use and also provide more ways of exploring and viewing our data. We describe these in the sections below.

### Protein viewer

**Figure 2.**
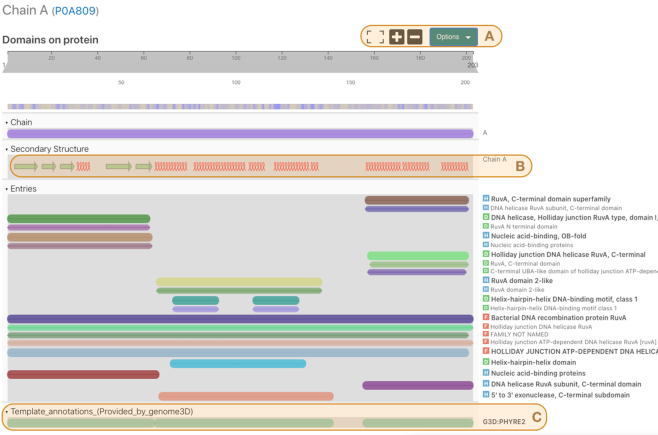
The InterPro protein viewer for the structure PDB:1CUK chain A of *E. coli* protein RuvA. Three sections of the image are highlighted, (**A**) viewer options, (**B**) secondary structure track and (**C**) Genome3D annotations.

The protein viewer is a key component of the InterPro website. It shows a graphical summary of the locations of entries and other relevant data for a single protein or structure chain (Figure 2). The protein viewer component is used within different parts of the website and is customised to display information depending on the page being viewed. For example, the protein viewer displays the protein and all matching InterPro and member database entries when part of a Protein page whilst the locations of chains and secondary structures are added to the viewer when part of a Structure page. The InterPro protein viewer was built by adapting the web components from the Nightingale project (24), which is an ongoing collaboration with other groups at EMBL-EBI, with the aim of producing a library of bioinformatics web components (https://ebi-webcomponents.github.io/nightingale/).

The three sections highlighted in Figure 2 show some recent developments in our protein viewer: Section A shows the option controls which allow users to select information such as colour scheme, track labels and tooltip behaviour in the viewer. There are also options to download an image of the viewer in PNG format or send a high resolution image to a printer or PDF file. Section B shows a track displaying regions of secondary structure predicted within the protein. Alpha helices and beta sheets are shown as helical loops and arrows respectively. The tracks in section C display information about predicted structures taken from Genome3D annotations. This is one of three places where we have enriched our data with Genome3D annotations; we have also added similar tracks for predicted structures in the protein page (e.g. https://www.ebi.ac.uk/interpro/protein/UniProt/P0A809/) and a new section in InterPro entry pages (e.g. https://www.ebi.ac.uk/interpro/entry/InterPro/IPR000085/genome3d/). These sections are the outcome of a collaboration with the Genome3D project (25).

**Figure 3.**
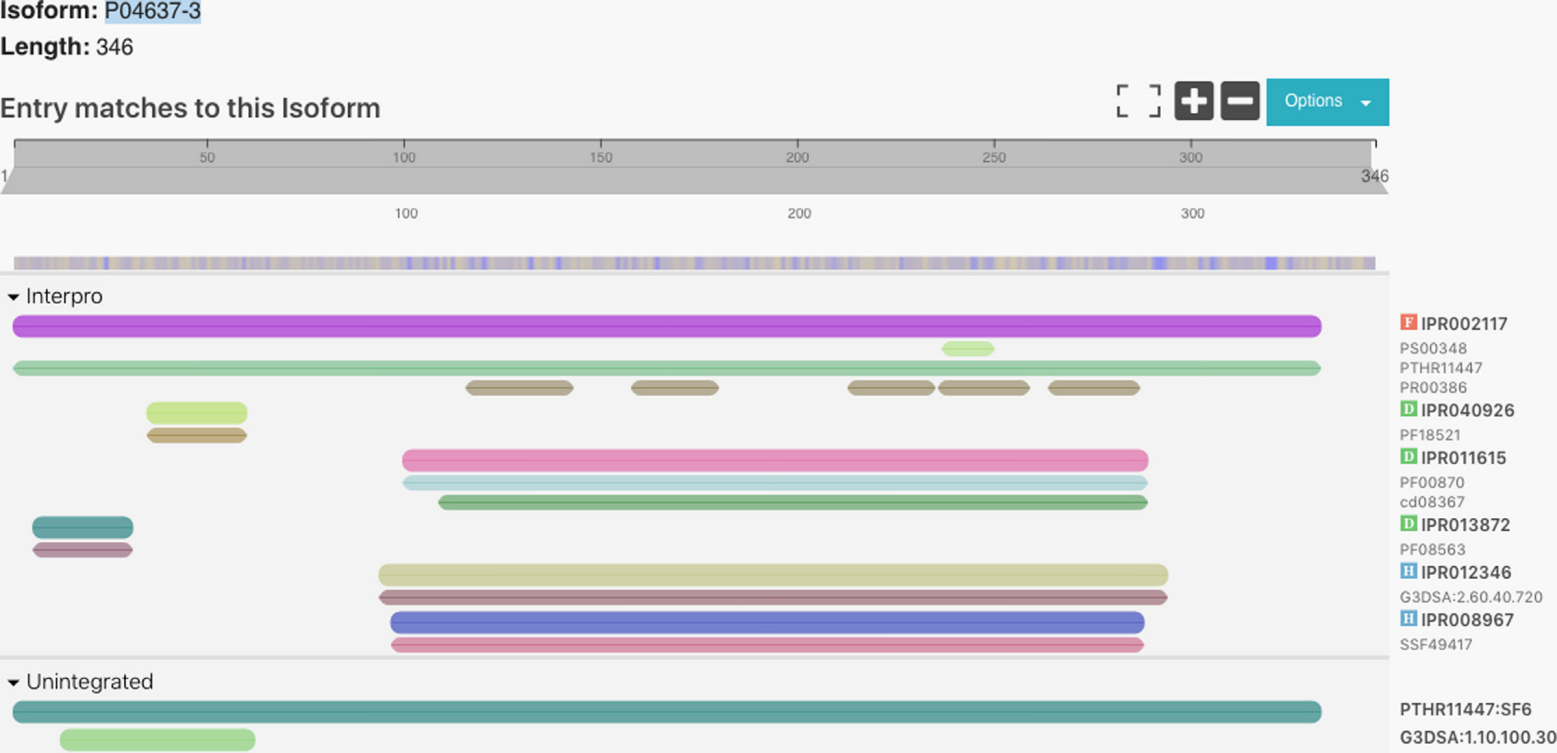
The InterPro protein viewer for the isoform P04637-3 of protein P04637.

Another new feature added to the InterPro website is the ability to view data for isoforms of a protein. This option is displayed in the protein pages. For example, the protein P04637 has nine isoforms (https://www.ebi.ac.uk/interpro/protein/reviewed/P04637/) which can be selected from the dropdown control shown in Figure 3. If the user selects one of them, a second protein viewer will be included in the same page so the user can compare the matches of the consensus protein with those in its isoform.

### Pfam alignments in InterPro

**Figure 4.**
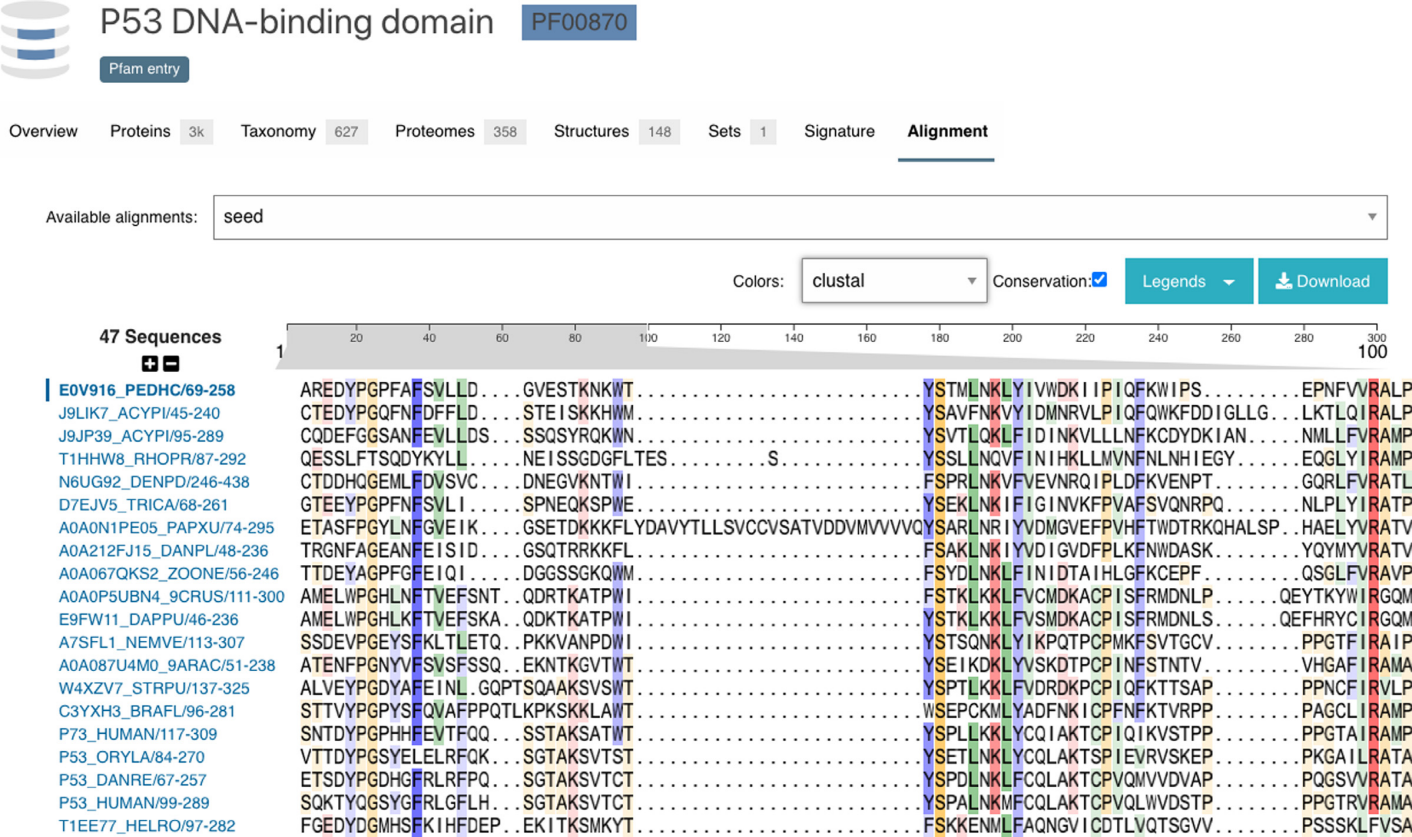
The InterPro multiple sequence alignment viewer for the P53 DNA-binding domain (https://www.ebi.ac.uk/interpro/entry/pfam/PF00870/entry_alignments/).

With the release of InterPro 80.0, we have added a viewer for multiple sequence alignments of Pfam entries. Figure 4 shows the Pfam seed alignment for the P53 DNA-binding domain (https://www.ebi.ac.uk/interpro/entry/pfam/PF00870/entry_alignments/?type=seed). We currently include the seed, full, uniprot and representative proteome alignments in this view. The responsiveness of the viewer depends on the length and number of sequences in the alignment as well as available memory and cpu performance on the client machine. Our testing has shown that the viewer can work well displaying alignments of up to 10 000 sequences. Alignment files are also made available for download, so users can view them in their tool of choice. The web viewer allows users to select colour schemes from a list that includes some used in popular alignment tools such as JalView or Clustal. Additionally, there is an option to display conservation information by tuning the opacity of the chosen colour for each amino acid residue based on the conservation of the residue in that alignment position. The viewer has been published as a web component in nightingale (https://ebi-webcomponents.github.io/nightingale/#/msa) and uses an up-to-date refactored version of MSAViewer (26).

### InterPro domain architecture search

The InterPro Domain Architecture Search feature has been redesigned. This tool enables users to identify proteins of interest based on the presence and order of particular domains. At the core of the new version is a text search engine supported by elasticsearch. We have simplified our representation of InterPro domain architecture (IDA) as a list of domains matching a protein sorted by location.

This structured text representation enables simple querying via the text search engine. Performing a query is now as simple as filtering for strings that contain the accessions of interest, in a given order. However, this requires that domains are ordered in a deterministic manner. This is a complex process for overlapping domains found across the many InterPro member databases. In order to overcome this difficulty we have taken the decision to limit the IDA definition to matches with Pfam entries because they do not overlap with each other.

The limitation of this approach is that proteins without pfam matches will not have an IDA string, even if they have matches with entries defined in other member databases. However, this change was necessary because searching through and calculating domain architectures across all member databases had become unscalable. The new method has vastly improved the performance of the search system. In our tests, queries to the new system are roughly an order of magnitude faster than the previous approach. Additionally, we have also cut down the time dedicated to IDA calculations in our release procedures by half.

**Figure 5.**
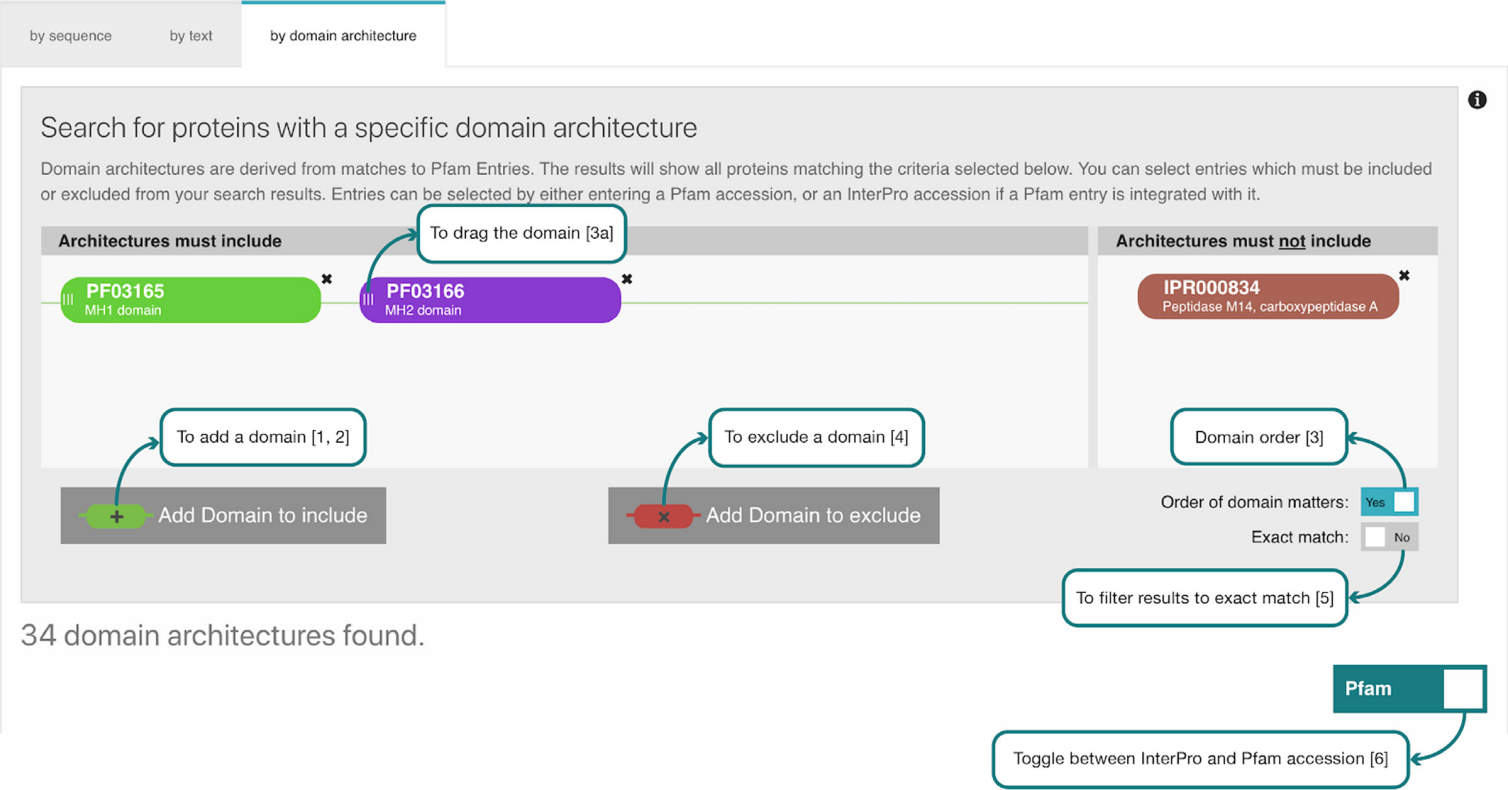
The InterPro Domain Architecture search interface.

These significant performance improvements allow us to create a more responsive and interactive web tool shown in Figure 5. This interface allows one or more domains to be searched in the query (1, 2) with the option of filtering results to show only results matching the order of the selected domains (3). The results can also be filtered to exclude domains (4) or to show architectures containing only the selected domains (5). The results can be viewed as InterPro or Pfam accession identifiers (6) because of the way we now base the IDA string on Pfam entries.

### InterProScan

Sequence search in InterPro is powered by the InterProScan software. InterProScan takes protein or nucleic acid sequences and searches them against InterPro's predictive models, which are provided by its member databases. The performance of InterProScan is largely dependent on the underlying performance of the individual binaries used for each member database as well as on the memory and CPU usage.

In the past years, we have worked to improve InterProScan's performance. For a long time, InterProScan has been limited in the number of input sequences it can handle efficiently. This was mainly due to higher memory usage resulting from the large number of analyses (21 analyses) that are carried out. Furthermore, there were some parts of processing, like the initial steps that included fetching the pre-calculated matches, which were single threaded. Refactoring these parts to include multithreaded solutions and improvements to memory handling resulted in improved performances. For example, until v5.36–75 running InterProScan against the Arabidopsis proteome (31 819 protein sequences) without using the match lookup service was taking 41 h. In version v5.45–80 (June 2020) and newer this analysis takes only 12 h. The performance gain is significant and increases with the number of input sequences, as shown in Table 3.

**Table 3.** Comparison of InterProScan runtimes for three key species, with and without using the pre-calculated match lookup service

		Runtime (h)			
		Lookup service: off		Lookup service: on	
Proteome	Number of protein sequences	v5.36–75	v5.45–80	v5.36–75	v5.45–80
Arabidopsis	31 819	41	12	30	0.7
Bonobo	42 997	144	15	151	0.7
Human	74 748	301	21	168	0.8

As part of the regular release procedure used to generate the InterPro database, matches are calculated for all UniParc protein sequences. These pre-calculated matches are made available to InterProScan via a lookup web service, to prevent unnecessary and wasteful recalculation. We have made improvements to the lookup web service on the backend as well on the client side. The data in the match lookup database is now more compact and therefore it is faster to get query results. The client has also been updated to use multithreading and is decoupled from the initial sequence loading steps that were a bottleneck to faster searches. This has resulted in a hundred fold improvement in performance. For example, the analysis of the human proteome with over 74 000 proteins (including isoforms) used to take at least 168 hours using the lookup match service, and it now only takes 45 min.

## DISCUSSION

Protein family and domain databases have retained a crucial position in the ecology of computational biology tools and resources. They are as important today as when they were introduced >20 years ago. Unfortunately, the available funding has not grown in line with the growth of sequence and annotation information and it has been challenging for the various resources to raise funding. This has meant that not all the resources are able to make regular updates and add new data. In the case of PRODOM, a fully automated domain database, we removed it from InterPro after it had not made an update for some years and the majority of its content was superseded by other resources. However, for both the PRINTS and SFLD resources the lead investigators retired and developments were ceased. In these two cases, InterPro provides a long-term sustainable mechanism for the data to be disseminated and updated to a limited extent. In the coming years we may see more such cases and thus InterPro may play a growing role in long-term database sustainability.

**Figure 6.**
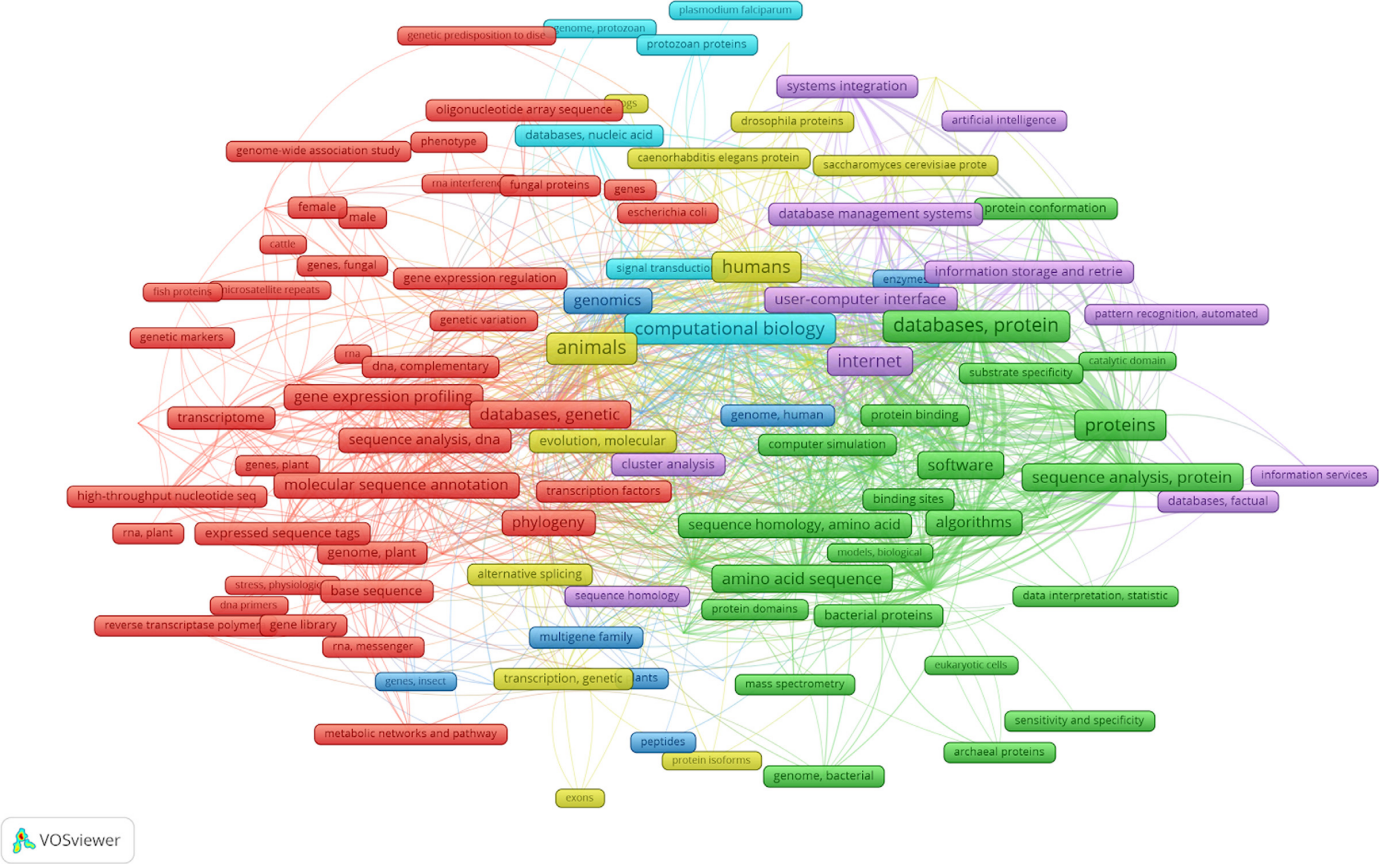
Mesh keyword network for papers mentioning InterPro. The image was generated with VOSviewer 27 using the Europe PubMedCentral API option to search for papers that mention InterPro within the title or abstract. Mesh keywords must be mentioned at least four times in the 426 papers matched to be included in the network.

InterPro is very widely used and cited across a range of different fields (see Figure 6). The major use cases are (i) to identify what protein family a protein belongs to, (ii) what protein domains, sites or other features a protein contains, (iii) to annotate a genome with protein family information, (iv) to annotate a genome with GO terms, (v) data reused within another database. Searching the full text literature at Europe PubmedCentral we find, 47% (4550 out of 9960 papers) of mentions of InterPro are found in the Methods section of the manuscripts, while 35% (3408 out of 9960) are found in the results section.

Running InterPro is a computationally intensive activity and thus will have environmental impacts through carbon dioxide emissions. We have continued to increase the efficiency of our InterProScan software so that despite growth in the number of sequences searched and the number of database signatures searched we can continue to reduce the environmental impact of our overall compute. In addition, the large scale reuse of InterPro data by other tools and resources helps to reduce the amount of redundant calculations that are made.

We welcome feedback from the community on any aspect of InterPro. Questions and remarks can be sent to the InterPro helpdesk (interhelp@ebi.ac.uk). Users may contribute to InterPro content by clicking the ‘add you annotation’ button on an entry page, and submit relevant biological information (e.g. literature references). Finally, InterProScan suggestions/feetbacks/issues can be reported on GitHub (https://github.com/ebi-pf-team/interproscan/issues).

## Data Availability

All data is freely available for browsing and download via the InterPro website https://www.ebi.ac.uk/interpro/.
